# Notes on Preparations for the Sick

**Published:** 1908-09

**Authors:** 


### Notes on Preparations for the Sick.

---

**Mostelle—Pure Juice of the Grape.**—The Grape Juice Co., Ltd., London and Tarragona.—This appears to be a very successful attempt to preserve ripe grape juice in its natural state without fermentation or the addition of foreign substances. The method of preservation is simply that of Pastuerisation: accordingly the wine should be consumed within four or five days after air has been admitted by the withdrawal of the cork. There are three varieties, Red, White and Golden Muscat; either of these when diluted with aerated water makes a refreshing, palatable and wholesome beverage. The wine is grown in the Province of Catalonia, in the north-eastern part of Spain. It is of a rich quality, containing some 25 per cent. of grape sugar. Accordingly it has an appreciable food value, may be given to children, and to all patients when an antiscorbutic fruit diet is desirable and fresh fruit may be unattainable or undesirable.

---

**Sterilla Soap:** an improved antiseptic liquid soap for surgical purposes.—Harold E. Matthews, Clifton.—Ether soaps have long been in use, but the odour of the ether is sometimes undesirable. This sterilla solution is practically odourless, and is most efficient for all purposes when soap is required. It mixes readily, does not corrode instruments, is useful for filming mirrors, and at the same time it sterilises them, as it is more potent than carbolic acid as a germicide. It must prove a safeguard against infection of skin and hair, and is an excellent protection against the traumatisms and inoculation of insects. It is the most satisfactory liquid soap which has come to our notice, both as regards personal hygiene and as a cleansing agent for surgical purposes.

**Staphylococcus Vaccine.**—Parke, Davis & Co., London—This is one of a series of bacterial vaccines now being produced in the Inoculation Department of St. Mary's Hospital, London, under the direction of Sir Almroth E. Wright. The flask contains one c.c., representing one hundred million of various strains of staphylococci applicable to the treatment of ordinary furunculosis, suppurating acne, staphylococci sycosis and other forms of staphylococcus infection. Other vaccines are the streptococcus from cases of erysipelas, the gonococcus, the tubercle, and the typhoid vaccine. Messrs. Parke, Davis & Co. have been appointed sole agents for the sale of these vaccines. Full instructions for use are given with each flask.

---

**" Vaporole " Aromatic Ammonia.**—Burroughs, Wellcome & Co., London.—A convenient means of instantly obtaining ammonia for inhalation in cases of fainting. These " Vaporoles " contain a solution of ammonia gas with aromatics ready for immediate use in emergency. They are more reliable than ordinary smelling-salts, which rapidly deteriorate. Each " Vaporole " contains a convenient quantity of solution in a capsule surrounded by absorbent material, the whole being enclosed in a covering of silk. When required the capsule is easily fractured by pressure between the fingers, and the vapour may be inhaled in the ordinary way. They are very efficient.

---

**Palatinoid Triple Valerianates.**—Oppenheimer, Son & Co., London.—This combination of valerian with zinc, quinine and iron should be most useful for the treatment of neurotic and other conditions (mostly in women) when the valerian preparations are indicated. They are odourless and unobjectionable. If valerian be necessary, surely such a preparation as this must be the most effective form of it, as it has none of the disagreeable qualities of the ordinary preparations.

---

**Ceredin Tablets : the Fat of Yeast.**—Bœhringer and Sœhne, Mannheim.—Each tablet contains ½ grain of ceredin and 3½ grains of sugar of milk. These tablets have obtained some reputation in the treatment of furunculi, suppurating acne, and such conditions, but in competition with the staphylococcus vaccine it seems doubtful if their reputation will be maintained. We have tried them in the carbuncles of diabetes, but with no very obvious result.

---

**Coryfin :** a new ethylglycollic menthol ester, designed to replace menthol as having a more prolonged action ; an external anodyne.—The Bayer Co., Ltd., London.—1. In nervous headaches coryfin should be lightly rubbed on the forehead, or it may be applied with a brush, which generally produces the desired result within ten to fifteen minutes. 2. (*a*) In common colds, intense coryza may be treated by dabbing the mucous membrane of the nose with a cotton pad or camel-hair brush moistened with coryfin. It is a good plan to leave a small plug moistened with coryfin in the lower part of the nostril and to endeavour to breathe through the nose. During long continued marches on the dusty road occasional moistening of the mucous membrane of the nose with coryfin relieves the respirations and prevents dryness in the nose and throat and its attendant discomfort. (*b*) In hoarseness or catarrhal irritation of the throat gargle with tepid water containing a few drops of coryfin, or allow a piece of sugar with three or four drops of coryfin to dissolve in the mouth.

---

**Bornyval.**—J. D. Riedel Co., London, E.C.—These elegant and inodorous gelatine pearls contain four grains of the active principals of valerian root. They should be of much value in cases of the various neuroses in which valerian is indicated, and in which this drug is becoming increasingly in demand.

**Gonosan.**—These capsules contain the active principals of kava-kava dissolved in pure East India sandal-wood oil. They are proving themselves highly useful in rapidly converting the purulent discharge into a mucus one, and so allaying the severe symptoms of the first stage.

